# Rare Case of Focal Gigantism of the Foot

**DOI:** 10.7759/cureus.43173

**Published:** 2023-08-08

**Authors:** Sri Arun Sellvam, Shashank Raghu, Juzaily F Leong, Firdaus Hafni, Rizal Abdul Rani

**Affiliations:** 1 Orthopaedics and Traumatology, Universiti Kebangsaan Malaysia Medical Centre, Kuala Lumpur, MYS; 2 Orthopaedics and Traumatology, Hospital Pulau Pinang, Penang, MYS; 3 Orthopaedics and Traumatology, Hospital Canselor Tuanku Muhriz, Kuala Lumpur, MYS; 4 Orthopaedics and Traumatology, Faculty of Medicine, Universiti Kebangsaan Malaysia Medical Centre, Kuala Lumpur, MYS

**Keywords:** macrodystrophia lipomatosa (mdl), congenital foot deformities, reconstructive surgery, localised gigantism, phenotype

## Abstract

Macrodystrophia lipomatosa (MDL) is a rare congenital variant of focused gigantism that is non-hereditary. Typically, MDL presents with localized gigantism in either the hand or foot. In this case report, we present the unique instance of a 12-year-old girl who has experienced enlargement of the first and second toes on her right foot since birth. Plain radiographs and MRI findings revealed the accumulation of fatty tissue around the first and second toes, medial and lateral aspects of the first metatarsal, extending up to the medial plantar arch of the foot. To enhance foot functionality and alleviate any issues with wearing footwear, a successful reconstruction surgical intervention was performed. As a result, the patient can now wear shoes without any difficulties. MDL is a very uncommon kind of congenital localised gigantism, and surgical consultation is frequently performed for cosmetic reasons.

## Introduction

A rare congenital, non-hereditary variant of focused gigantism is known as macrodystrophia lipomatosa (MDL) [1]. Generally, localized gigantism of the hand or foot is a presenting symptom of MDL. The excessive growth of fibroadipose tissue is mostly composed of mesenchymal components, primarily the periosteum, bone marrow, nerve sheaths, or muscles [2,3]. Although the exact cause of bony expansion is unknown, the pathophysiology is assumed to be caused by the bone's endosteal and periosteal deposition [4]. In addition to clinical findings, imaging is crucial for diagnosis [2,4]. MDL patients generally receive surgical procedures for aesthetic rather than mechanical reasons.

## Case presentation

A 12-year-old female child was referred from a secondary centre with a history of enlargement of the first and second toes of the right foot since birth. The child was diagnosed at birth but has not been on follow-up since then. The child was brought by parents as the deformity was progressively increasing in size over the years causing difficulty in attaining proper footwear, leading to troubled ambulation. However, the child did not complain of any pain during ambulation even for long distances or climbing up and down the stairs.

This child is the third child of four siblings in a non-consanguineous marriage. She was born via spontaneous vaginal delivery, and there is no significant prenatal or perinatal history. No family history of similar presentation or any other similar manifestation or congenital anomalies in other siblings.

**Figure 1:**
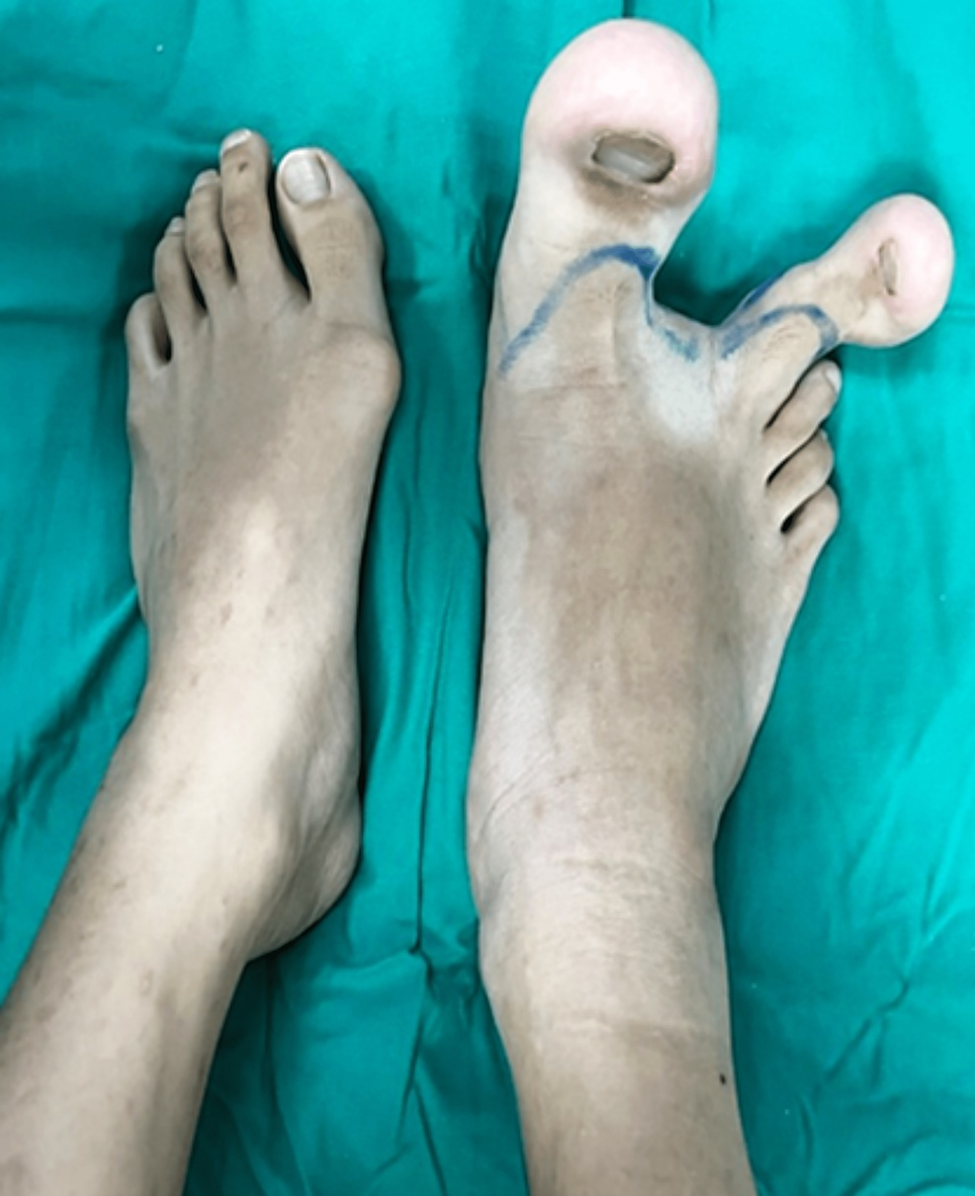
Clinical photograph of each foot demonstrates the macrodactyly involving the right first and second toes with dorsal angulation of both toes.

On examination, the right foot's first and second toes are evidently enlarged, with the first toe appearing to be bigger than the second toe. The rest of the other toes appear as normal-sized as the left foot. Both the toes grew in a divergent fashion with massive soft tissue overgrowth over the plantar aspect (Figure 1). Neurovascular status was intact, and there are no skin colour changes, ulceration, palpable thrill, or pigmentation over both toes. The rest of the right foot appearance and physical examination were normal. On further examination, no cutaneous manifestation of neurofibromatosis was seen.

**Figure 2:**
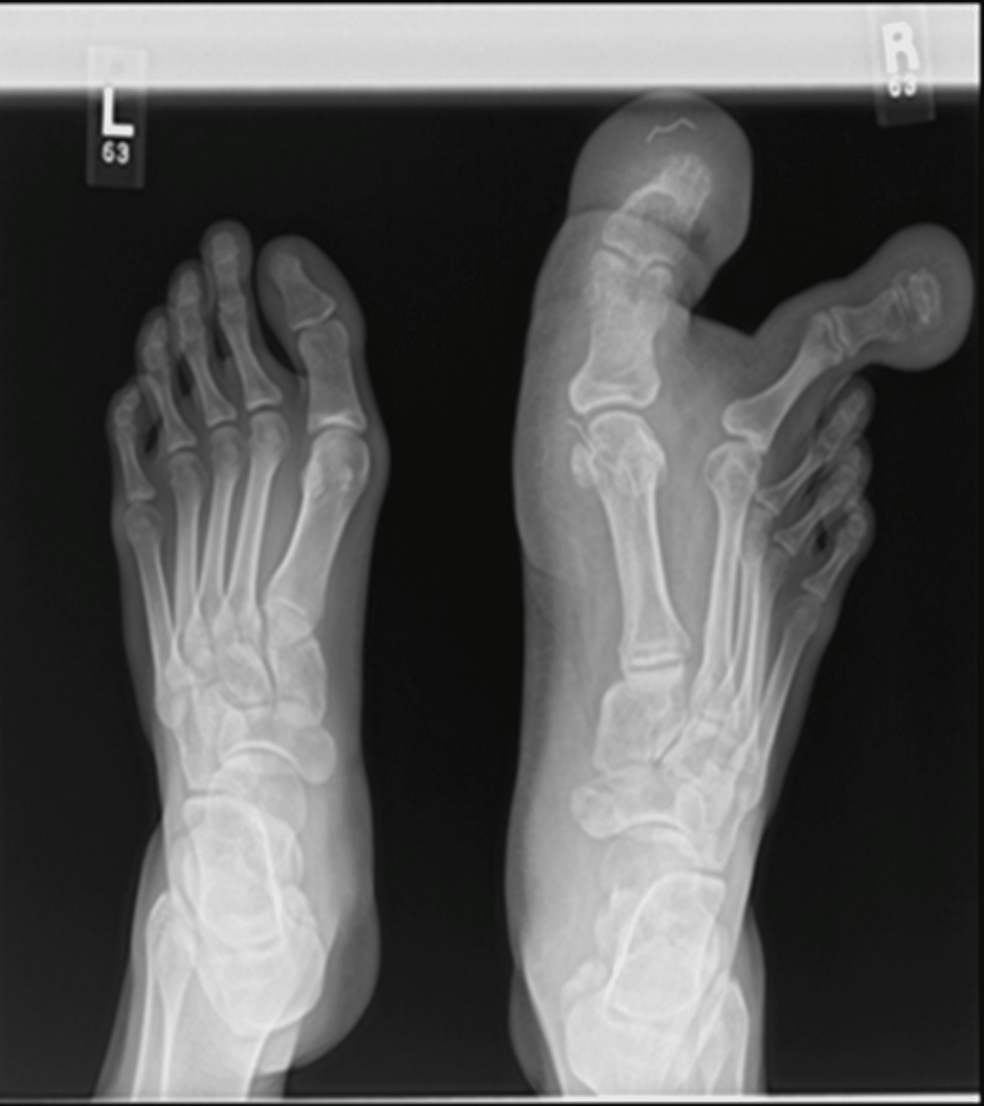
Plain radiograph of both feet shows soft tissue swelling on the plantar aspect of the affected toes and enlarged first and second metatarsals, and all the phalanges of both involved toes with splaying of the phalanges.

Radiographical imaging showed large first and second metatarsals, along with large all phalanges of the first and second toes. There was a marked increase in the soft tissue surrounding both toes, with a lateral view of the imaging showing gross soft tissue overgrowth over the plantar aspect (Figure 2).

**Figure 3:**
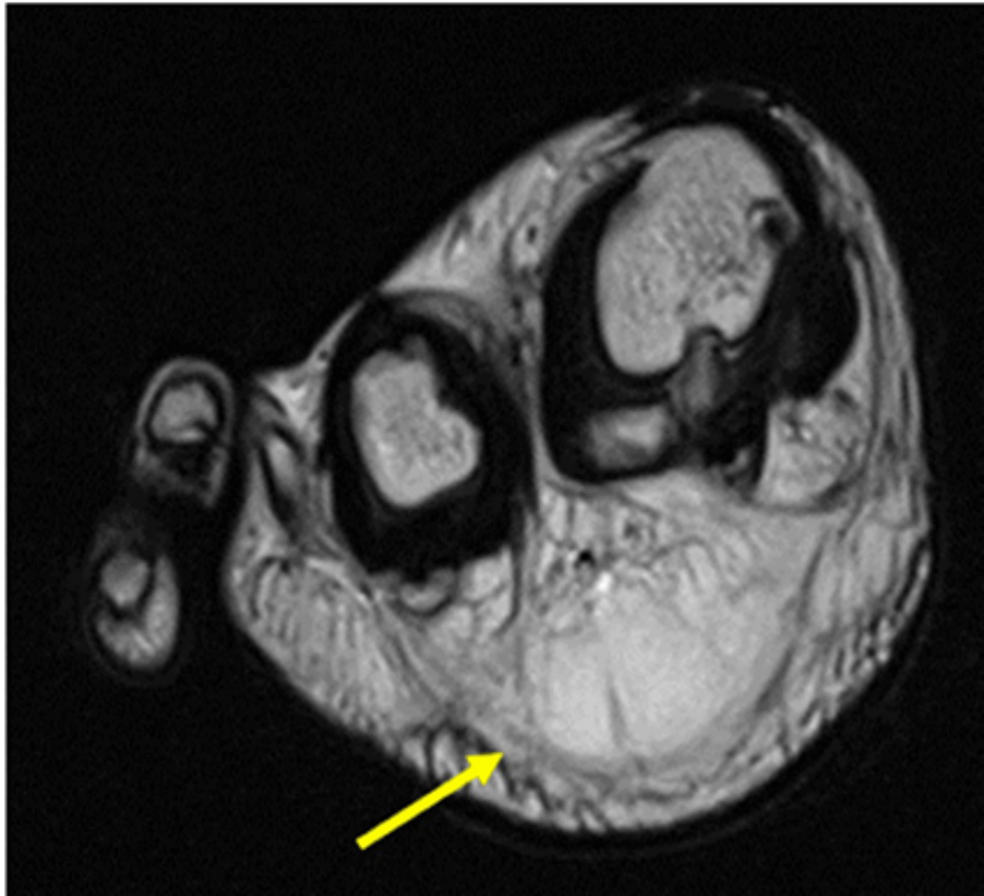
Axial MRI fat-suppressed image shows the plantar lesion with similar homogeneity to normal fat tissues.

**Figure 4:**
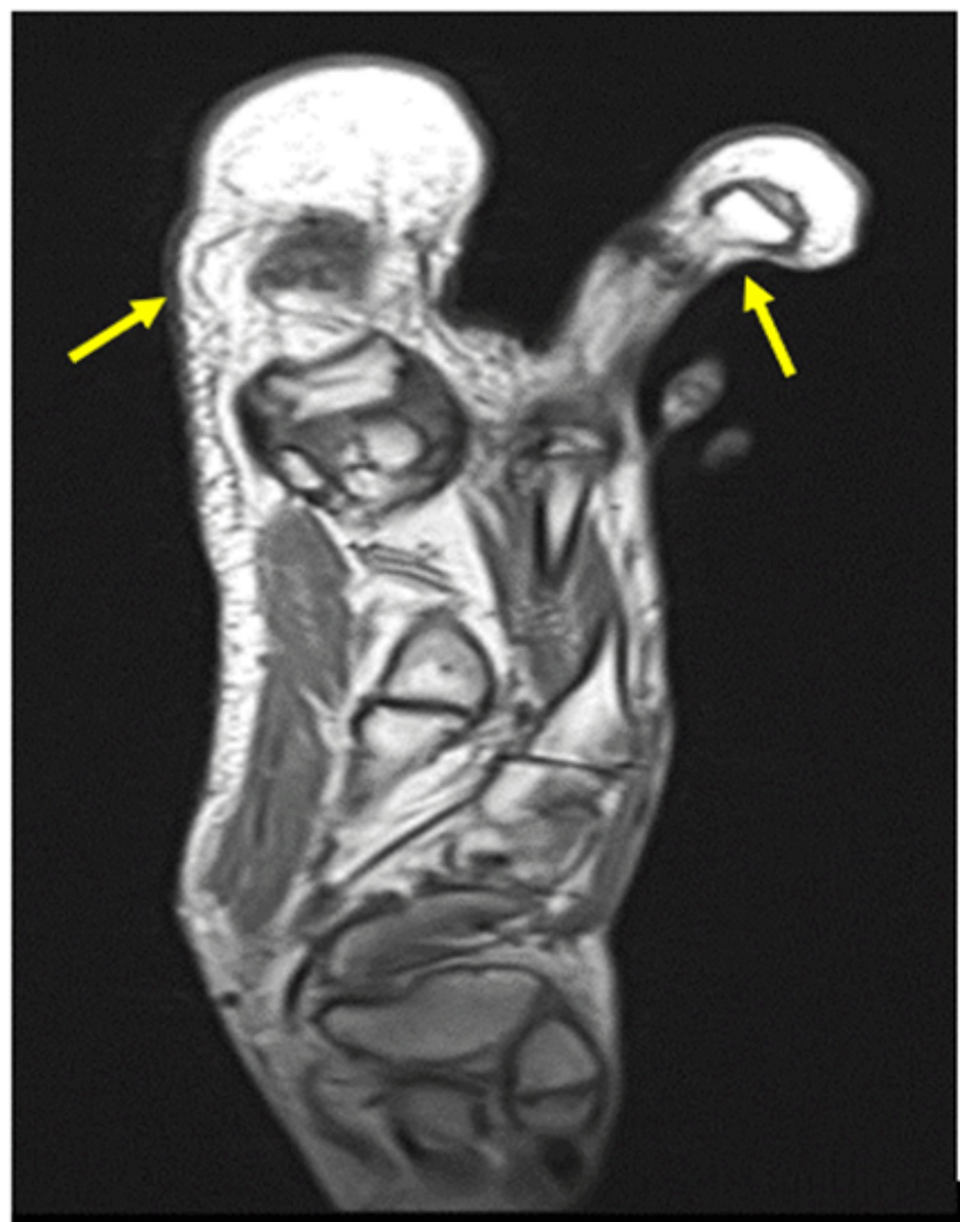
Sagittal MRI image reveals the proliferation of fatty tissue on the first and second toes with signal intensity similar to that of subcutaneous fat.

The MRI of the right foot showed bone and soft tissue enlargement of the first and second toes. There is an accumulation of fat tissue with no discernible capsule around the first and second toes, medial and lateral aspects of the first metatarsal with extension up to the medial plantar arch of the foot. A soft tissue lesion was noted deep to the flexor hallucis longus, involving the lateral digital nerve of the first toe. There was no evidence of infiltration to adjacent bone and joint seen, and no features of vascular malformation were seen. The flexor and extensor tendons of the foot appear intact. The MRI findings are reported as macrodactyly lipomatosa (Figures 3-4).

**Figure 5:**
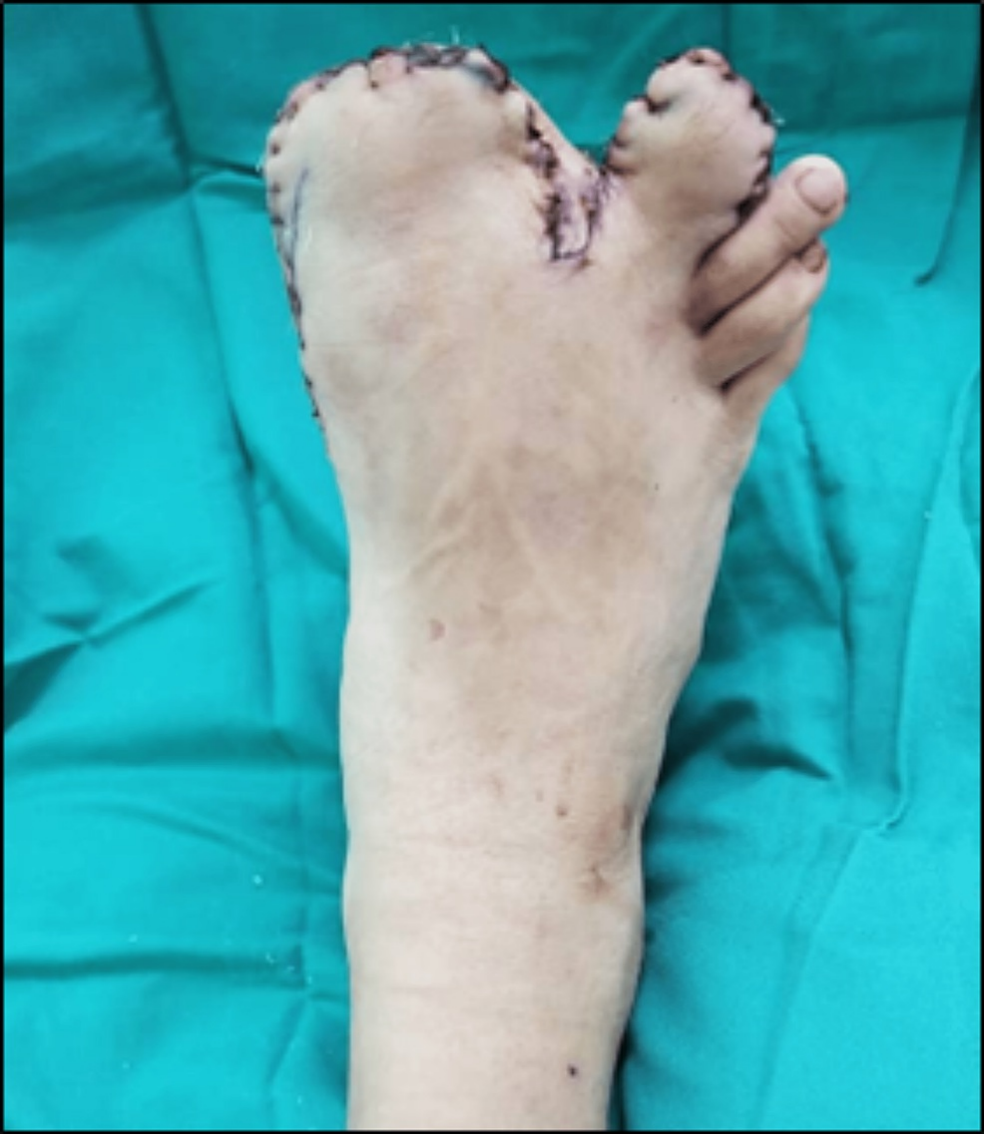
Photograph of post-operative disarticulation at the proximal phalanx level and reconstruction of the right first and second toes with the creation of the first webspace.

A preoperative incisional biopsy was not done for this patient, as no evidence of malignancy was seen. Right first toe and second toe disarticulation and reconstruction were done through fish mouth incisions made along the first and second toe proximal interphalangeal joint (PIPJ), extended into the first webspace. The proximal phalanx of both the first and second toes refashioned with flexor and extensor tendons is anchored to the proximal phalanx. Excessive fat tissue over the plantar aspect was excised, and satisfactory wound closure was achieved (Figure 5).

**Figure 6:**
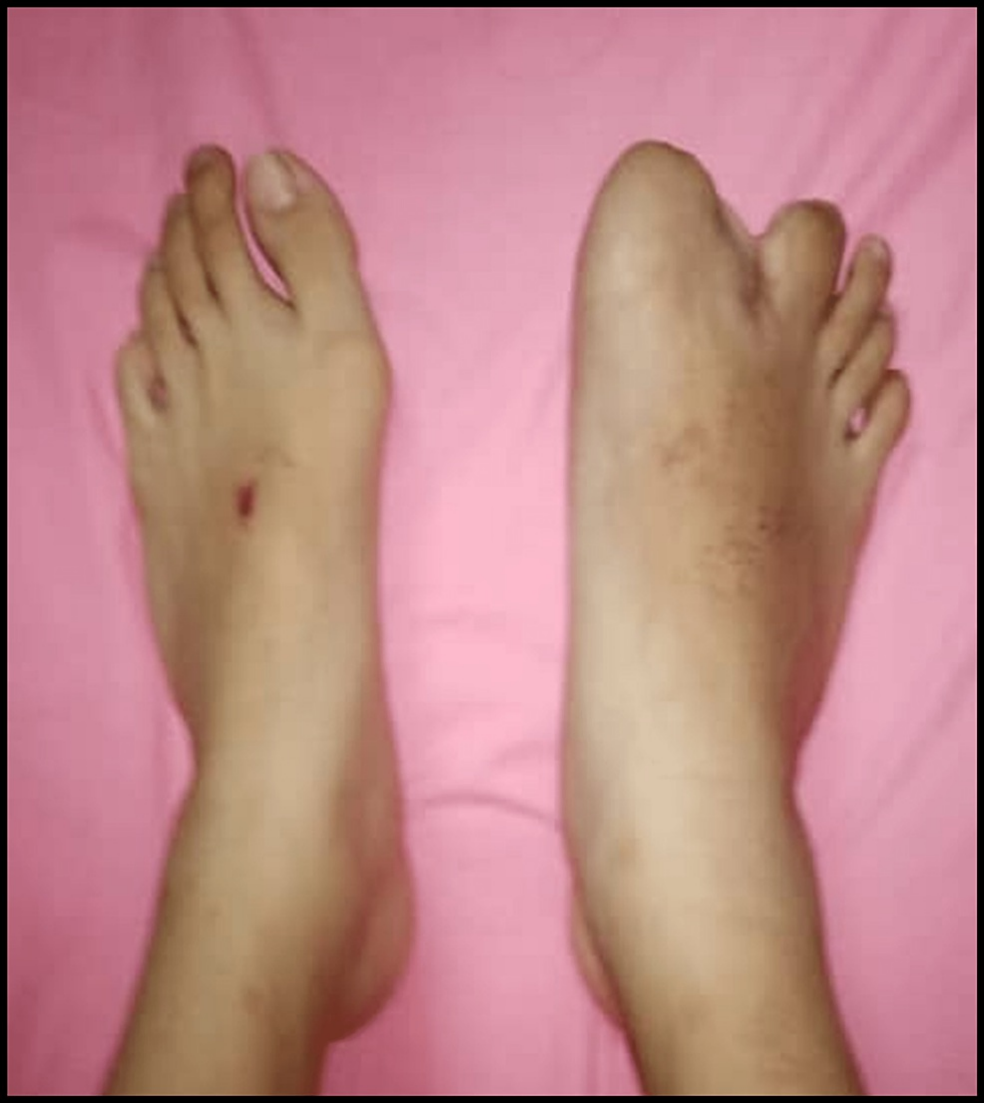
Photograph of post-operative at six months.

Histopathological examination showed lobular proliferation of mature adipocytes separated by thick fibrous septae. No lipoblast, stromal atypia, or other types of evidence of malignancy was seen. A benign proliferation of fibrofatty tissue was consistent with macrodactyly. Upon further follow-up at the clinic, the wound healed well over the right foot's first and second toes. There is no numbness or discolouration over the toes or foot, indicating a good neurovascular status (Figure 6). The child was able to accommodate into suitable covered footwear and able to ambulate without pain.

## Discussion

In 1925, Feriz introduced the term macrodystrophia lipomatosa (MDL), a rare congenital, non-hereditary variant of focused gigantism in the foot or hand. Feriz explained the term as an overgrowth of the unilateral lower limb [1,5]. By outlining two types of macrodactyly, Barsky created classifications for local gigantism. The two forms that are described are the static form, in which the growth of the larger digits increases proportionally to the growth of other body components, and the progressive form, in which the growth of the enlarged digits increases disproportionally to the growth of the body [2,6]. Fatty overgrowth is usually seen in the progressive form, similar to the term described by Feriz [4]. In our case, an MRI of the right foot revealed that the first and second toes' bones and soft tissues had become larger compared to the other toes. Around the first and second toes, adipose tissue has accumulated but with no visible capsule. Hence, Feriz's theory and Barsky's taxonomy of progressive form are in line with our case description.

Although MDL's etiopathogenesis is not fully understood, there are a number of theories that attempt to explain it [5]. Accordingly, theoretical explanations include lipomatous degeneration, abnormal foetal circulation, injury to the buds of the limbs, change in somatic cells throughout intrauterine life, and expansion of the pertinent nerve [7]. Despite the uncertainty surrounding the aetiology and pathophysiology of this disorder, a substantial cohort study on macrodactyly cases found a high positive rate of PIK3CA gene mutation in patients, which broadens our understanding of the molecular pathology underlying these congenital malformations [2,8]. In parallel with this, Wu et al. reported that, with the aid of Sanger and next-generation sequencing, the PIK3CA gene mutation that causes activation was found in the afflicted adipose, nerve, and skin tissues of 10 cases [3].

To the best of our knowledge, no published article has reported an exact incidence due to the disorder's rarity and various terms used to describe the disorder. In the distribution of MDL, men outnumber women by a large margin. Accordingly, Wu et al. [3] reported among 90 patients that there were more male patients than female ones. The distal and volar areas of the digits are more frequently involved. Lower limb involvements are 15 times more common than upper limb involvements [2]. This congenital defect is most frequently encountered in the distributions of the medial plantar nerves in the lower extremities, which typically include the first to third toes, and the median nerve in the upper extremity, which frequently presents with symptoms of carpal tunnel syndrome [4]. The child in our case had dorsal angulation of the affected toes due to significant fatty tissue deposition on the plantar aspect of the affected first and second toes of the right foot.

Generally, MDL patients usually seek medical attention later in life and more commonly for cosmetic than for mechanical problems. Surgery continues to be a major component of the treatment for macrodactyly [3]. Therefore, our patient sought surgical treatment since the growth of the affected digits made it difficult for her to locate shoes that fit. Enhancing physical appearance while retaining the most possible neurologic function is the main objective of surgery in the management of macrodactyly lipomatosa [1,5]. When development slows down after puberty, surgery is routinely performed. To have the greatest results, it is suggested that numerous debulking surgeries, epiphysiodesis, and different osteotomies be carefully and purposefully performed [3,4]. In our surgical procedure, we reconstruct both affected digits at the level of the proximal phalanges while keeping the first webspace for a more aesthetically pleasing appearance. Debulking is a procedure to remove extra fatty tissue from the plantar area of the foot in order to produce a flatter surface over the forefoot for evenly distributing weight over the right foot.

## Conclusions

In conclusion, MDL is a rare type of congenital localised gigantism, and surgical consultation is frequently requested for aesthetic reasons. Histopathology and imaging work together to confirm the diagnosis. Surgical treatment should be well-planned with the aid of imaging and tailored accordingly to each case for the best outcome in both cosmetic and functional requirements. A multidisciplinary approach and additional systematic research with more patients are needed to investigate the relationship between mutations and clinical phenotype and prognosis of the MDL disorder.
